# Feasibility, Reliability, and Validity of a Smartphone Based Application for the Assessment of Cognitive Function in the Elderly

**DOI:** 10.1371/journal.pone.0065925

**Published:** 2013-06-11

**Authors:** Robert M. Brouillette, Heather Foil, Stephanie Fontenot, Anthony Correro, Ray Allen, Corby K. Martin, Annadora J. Bruce-Keller, Jeffrey N. Keller

**Affiliations:** Pennington Biomedical Research Center/Louisiana State University System, Baton Rouge, Louisiana, United States of America; University Hospitals of Geneva, Switzerland

## Abstract

While considerable knowledge has been gained through the use of established cognitive and motor assessment tools, there is a considerable interest and need for the development of a battery of reliable and validated assessment tools that provide real-time and remote analysis of cognitive and motor function in the elderly. Smartphones appear to be an obvious choice for the development of these “next-generation” assessment tools for geriatric research, although to date no studies have reported on the use of smartphone-based applications for the study of cognition in the elderly. The primary focus of the current study was to assess the feasibility, reliability, and validity of a smartphone-based application for the assessment of cognitive function in the elderly. A total of 57 non-demented elderly individuals were administered a newly developed smartphone application-based Color-Shape Test (CST) in order to determine its utility in measuring cognitive processing speed in the elderly. Validity of this novel cognitive task was assessed by correlating performance on the CST with scores on widely accepted assessments of cognitive function. Scores on the CST were significantly correlated with global cognition (Mini-Mental State Exam: r = 0.515, p<0.0001) and multiple measures of processing speed and attention (Digit Span: r = 0.427, p<0.0001; Trail Making Test: r = −0.651, p<0.00001; Digit Symbol Test: r = 0.508, p<0.0001). The CST was not correlated with naming and verbal fluency tasks (Boston Naming Test, Vegetable/Animal Naming) or memory tasks (Logical Memory Test). Test re-test reliability was observed to be significant (r = 0.726; p = 0.02). Together, these data are the first to demonstrate the feasibility, reliability, and validity of using a smartphone-based application for the purpose of assessing cognitive function in the elderly. The importance of these findings for the establishment of smartphone-based assessment batteries of cognitive and motor function in the elderly is discussed.

## Introduction

The number of elderly individuals in the United States is growing at an unprecedented rate, with nearly 10,000 individuals a day turning 65 in the United States [1]. This rapid expansion in the number of elderly is also being accompanied by an ever increasing demand for healthcare needs in the geriatric population [1]. Finding strategies that allow for the most efficient mechanism(s) of monitoring the health status of the elderly and identifying the most efficient mechanism(s) for the delivery of remote health surveillance, disease prevention, and medical support will be crucial to adequately addressing the healthcare needs of the elderly in the coming years. Recent technological advances such as telehealth and telemedicine products have proven to be very useful in a variety of settings [2]–[6]. Each of these mechanisms promotes remote patient, physician, and/or student interactions, which are critical features for the next generation of clinical research/care [2]–[8].

Smartphones are specialized mobile phones, which contain additional computing capacity/capabilities, that are likely going to be the principal platforms for the development of the next generation of clinical care/research [9], [10]. In addition to being mobile, and increasingly ubiquitous in the general population, smartphones have an ever increasing number of medical-related applications which can potentially be used for clinical research/care. Several recent papers have described the potential for smartphone-based applications to assist in a wide range of clinical research areas and the delivery of medical care related to falls, medication adherence, food intake, depression, stroke, diabetes management, rehabilitation, medication adherence, and patient point-of-care services [9]–[18].

Despite the numerous advances made in conducting smartphone research, almost nothing is known in terms of the feasibility and validity of using smartphone-based technologies for clinical research/interventions in the elderly. Development of smartphone-based assessments for cognition, mobility, and frailty research in the geriatric population is a crucial area of current research. The goal of the current study was to develop a new cognitive assessment test (processing speed) utilizing touch screen technology, with remote testing capabilities, that is suitable for use in an elderly non-demented population. In this paper, we report for the first time on the development of a new smartphone-based application to measure cognitive function in the elderly. This Color-Shape Test (CST) was found to be a reliable and valid tool for the measurement of cognitive processing and attention in the elderly. The importance of this finding to the development of smartphone-based assessment batteries for cognitive and motor function in the geriatric population is discussed.

## Methods

### Participants

All 57 participants for this study were recruited from the Louisiana Aging Brain Study (LABrainS) [19]–[24]. Volunteers for the LABrainS study are recruited through regular outreach efforts of the Institute for Dementia Research and Prevention (IDRP) throughout Louisiana, with LABrainS participants consisting of non-demented elderly individuals willing to undergo annual cognitive assessments. The cognitive assessments for these participants are conducted utilizing the Uniform Data Set (UDS) neuropsychological battery established by the National Alzheimer’s Coordinating Center (NACC). Participants in LABrainS are individuals over the age of 60 with no existing diagnosis of dementia or cognitive impairment. All participants have a Clinical Dementia Rating (CDR) of 0 and a Mini-Mental State Exam >25, consistent with the absence of dementia. Additionally, they have no history of other neurological condition (e.g., cerebrovascular disease, Parkinson’s disease, brain injury) that could cause cognitive sequelae. Individuals for this study used the smartphone provided by IDRP staff and had a wide range of experience in smartphone usage ranging from no previous experience to daily use of a smartphone.

### Equipment

The assessment software was developed specifically for use with touch screen smartphones and was compatible with all Apple iPhones and Android phones. The software used for development was a Microsoft ASP.Net application which was programmed using C#, jQuery Mobile, and javaScript. Data from test administrations were captured using a Microsoft SQL Server database along with a time stamp indicating when the data were recorded.

### Measures

#### a. Uniform data set

The neuropsychological test battery from the Uniform Data Set (UDS) of the Alzheimer’s Disease Centers’ (ADC) program of the National Institute on Aging consists of brief measures of attention, processing speed, executive function, episodic memory, and language [25]. The battery consists of measures of dementia severity (Mini-Mental Status Examination) [26], attention (Digit Span Forward and Backward from the Wechsler Memory Scale-Revised) [27], processing speed (Digit Symbol Subtest from the Wechsler Adult Intelligence Scale-Revised [28], Trails Making Test Part A), executive function (Trails Making Test Part B [29]), memory (Logical Memory Story A Immediate Recall from the Wechsler Memory Scale-Revised, Logical Memory Story A Delayed Recall from the Wechsler Memory Scale-Revised [29]), Verbal Fluency (Animal List Generation [30] and Vegetable List Generation), and naming (Boston Naming Test - 30 odd items [31]). In addition to the UDS neuropsychological test battery, participants were also administered the Geriatric Depression Scale (GDS, 15-item version) [32] as a measure of depressive symptomatology.

#### b. Color-Shape test

**Figure 1:**
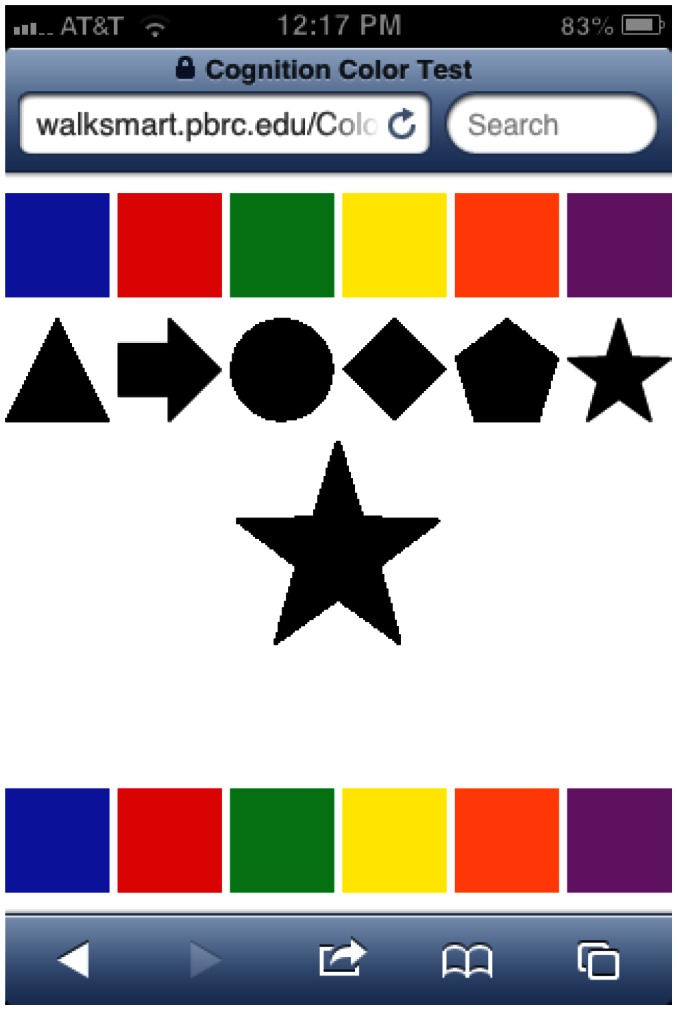
Screen shot of Color-Shape Test. A representative image of the screen shot for the Color-Shape Test is presented.

As part of their annual visit, study participants were administered the Color-Shape Test (CST), a test of cognitive processing speed designed at Pennington Biomedical Research Center. The test design is as follows: The test interface (see figure 1) consists of paired colors and shapes which appear at the top of the screen and serve as a “legend” for the task. At the bottom of the screen are colored “pads” which correspond to colors in the legend. Participants use these pads to respond. When the task is initiated, random shapes from the legend appear one at a time in the center of the screen. The shape remains on the screen until the subject taps the color pad (at the bottom of the screen) that corresponds to the one paired with the shape in the legend (top of the screen). Once a response (correct or not) is made, a new shape is presented and the task repeats. Participants were allowed a 30-second practice test prior to the initiation of the actual evaluation. The actual test records the number of attempts and number correct over a 2-minute testing interval.

Following completion of the neuropsychological testing battery, the CST was administered to participants in the clinic using the examiner’s iPhone 4. The test was administered to each participant utilizing the following standardized instructions:

“This is a test of processing speed. When the test begins, you will see the following screen appear on the smartphone.”

Participants were then shown a screen shot of the interface to facilitate proper instruction of the task. Proper test-taking procedure was demonstrated on the screen shot along with the following verbal instructions:

“The object of this test is to correctly match as many shapes to their corresponding color as possible. Please try to respond as quickly as you can.”

Additionally, as a means of measuring reliability, participants who had been in possession of a smartphone for more than 6 months were emailed a link to the CST for re-administration 2 weeks later. Of the 57 participants who performed the clinic administration of CST, 18 were identified as having been in possession of their personal smartphone for at least 6 months. These 18 participants were emailed a link to retake the CST 2 weeks later. Of the 18 who were emailed, 11 responded by retaking the CST off-site under uncontrolled conditions on their personal phone.

## Results

### Participant Profile

A total of 57 volunteers from the longitudinal Louisiana Aging Brain Study (LABrainS) participated in this study. The demographics for the age, sex, and education of the participants are provided in Table 1. Additionally, the global cognitive status and GDS measures for the study group are provided in Table 1. Together, these data suggest that the study participants in the current study did not exhibit evidence for dementia or depression. The mean performance scores of study participants on all cognitive assessments are provided in Table 2.

**Table 1:** Subject demographics and characteristics.

Age	67.18 yr±1.02 yr
Sex	17M/40F
Education	16.37 yr±0.31
Uniform Data Set	2.10±0.05
Geriatric Depression Scale	1.16±0.18

All data presented as the mean and SEM.

**Table 2:** Profile of Cognitive Performance in Study Participants.

Color-Shape Test (Attempts)	63.77±2.35
Color-Shape Test (# Correct)	61.77±2.48
Mini-Mental State Exam	29.25±0.16
Logical Memory I	15.18±0.53
Logical Memory II	14.84±0.57
Digit Span Forward	7.63±0.34
Digit Span Backward	5.24±0.19
Digit Symbol	54.98±1.65
Trails Making Test A	29.65±1.78
Trails Making Test B	69.98±3.56
Boston Naming Test	28.89±0.16
Animal List Generation	23.89±0.63
Vegetable List Generation	16.54±0.58

All data presented as the mean and SEM.

### Reliability and Validity of the CST for Processing Speed

Study participants were analyzed for their ability to complete the CST smartphone application as described in the Methods section. Study participants exhibited a mean of 63.77±2.35 attempts on the task and answered correctly on 96.8% of the attempts. The test re-test reliability for the CST was observed to be significant (r = 0.726, p = 0.02). Re-tests were all conducted under uncontrolled off-site conditions. In order to begin validating the CST, we next examined the correlation between the CST and established measures of cognition including cognitive processing speed (Digit Symbol Test, Trails Making Test) (Table 3). In these analyses, we observed that the CST was significantly correlated with Digit Symbol Test (r = 0.508, p<0.00008), Trails Making Test A (r = –0.651, p<0.000001), and Trails Making Test B (r = −0.384, p<0.004). Measures of attention were also significantly correlated with the CST including the Digit Span Forward (r = 0.427, p<0.001) and Digit Span Backward (r = 0.434, p<0.001) tests. The CST was also found to be significantly correlated with the Mini-Mental State Exam (r = 0.515, p<0.00004). The CST was not correlated with naming or verbal fluency tasks (Boston Naming Test, Vegetable/Animal Naming) or memory tasks (Logical Memory Test) (Table 3). Age was significantly correlated with CST performance (r = −0.647, p<0.0000001) (Table 3). The CST was not correlated with education (Table 3).

**Table 3:** Correlation Between CST and Age, Education, Depression, and Established Cognitive Assessments.

Age	r = −0.647* (p<0.0000001)
Education	r = −0.124
Geriatric Depression Scale	r = 0.0005
Mini-Mental State Exam	r = 0.515* (p<0.00004)
Logical Memory I	r = 0.217
Logical Memory II	r = 0.145
Digit Span Forward	r = 0.427* (p<0.001)
Digit Span Backward	r = 0.434* (p<0.001)
Digit Symbol	r = 0.508* (p<0.00008)
Trails Making Test A	r = −0.651* (p<0.000001)
Trails Making Test B	r = −0.384* (p<0.004)
Boston Naming Test	r = 0.024
Animal List Generation	r = 0.147
Vegetable List Generation	r = 0.02

All data presented as the correlation between CST and individuals’ cognitive measures.

* Statistically significant correlations (p<0.05) based on Pearson correlation coefficient.

## Discussion

These data demonstrate the feasibility of using smartphone-based applications for the assessment of cognitive function in the elderly. Importantly, these data were collected in participants who had a range of experience with the use of smartphones, ranging from no experience to daily use of smartphones. While we did not collect data on the actual experience of individual study participants, we were aware of the range of experience based on statements made by the individuals ranging from no previous experience to those with extensive experience. Additionally, it is important to point out that the study was not designed to select for people with moderate to high proficiency with smartphone technology. Within this study sample, the CST was found to be a reliable and valid measure of cognitive function, supporting the concept that smartphone-based assessments are likely to provide a useful means of assessing cognition in the elderly, despite the relatively low use of smartphones in the geriatric population. While the relatively small screen of the smartphone was not an insurmountable obstacle to conducting assessments, it was clearly one of the largest experimental obstacles observed in the current study. The screen of the smartphone made for some inadvertent errors with regards to the color-shape matching, as well as premature screen advancement. It is likely that the use of tablet devices, which have a much larger screen and higher computing capacity, may be able to overcome some of the limitations associated with the smartphone use in the present study (i.e., larger surface for manipulating objects, larger screen to accommodate larger objects). Recent surveys indicate that elderly subjects purchase significantly more tablets as compared to smartphones, suggesting that tablet-based cognitive assessments may have a larger population base for conducting cognitive assessments/studies in the geriatric population.

Based on the design of the CST, we hypothesized that the CST would correlate significantly with other established measures of processing speed. The CST application in the current study was observed to be a reliable and valid measure of processing speed and attention in the elderly and suggested that this cognitive assessment tool may be a useful and mobile mechanism for assessing cognitive function in the elderly. The majority of previous studies developing computerized cognitive assessment tools have focused on the use of desktop-based technologies, despite the fact that desktop computers have multiple limitations that affect their potential usage for clinical care/research. For example, desktop computers do not lend themselves readily for mobile/real-time assessments and require dedicated space and Internet availability, both of which may limit patient access. Smartphones/tablets offer a nearly unlimited number of applications that can be seamlessly combined for the comprehensive assessment of cognition, health status, and mobility under free-living conditions. For example, studies have reported on the use of smartphones in a non-geriatric population for monitoring daily activities, rehabilitation, mobility, heart rate, glucose levels, blood pressure, and food intake [11]–[18]. The ability of smartphones to be worn on the body makes them particularly attractive for geriatric studies [9]–[11]. For example, by coupling existing smartphone-based clinical tools (i.e., EKG, EEG, accelerometer, blood pressure recorders) with smartphone-based measures of cognition, researchers could elucidate the interactions between changes in multiple health outcomes and cognitive decline. This real-time comprehensive assessment of overall health status in the elderly could rapidly transform geriatric research, particularly in the areas of dementia, falls, and frailty research. Studies are currently under way by our group to develop a comprehensive battery of cognitive assessments that can be delivered via smartphone/tablet-based technologies to monitor cognitive status in a longitudinal manner within the elderly. This cognitive status battery will then be coupled with additional smartphone-based clinical tools to determine the feasibility of using smartphones for the real-time assessment of frailty in the elderly.

In addition to monitoring the health status of elderly individuals, smartphone/tablet-based technologies may ultimately be used for the delivery and surveillance of clinical interventions in the elderly. For example, smartphone-based applications have been used to deliver clinical care in a non-geriatric population for a variety of conditions including rehabilitation, depression, and falls [6], [12], [15]. Smartphones were found to be an efficient means of delivering interventions, as well as to provide an effective means for monitoring adherence, and measuring outcomes, for each of the interventions. It is important to point out that smartphone-based studies will also likely transform the way individuals are recruited for clinical research trials by decreasing the impact of geography, clinical capacity, patient diversity, and recruitment costs [12]. Such advances will allow for more studies, larger studies, and more diverse forms of studies to be conducted by incorporating the use of smartphones in geriatric research.
